# Subjective stress, objective heart rate variability-based stress, and recovery on workdays among overweight and psychologically distressed individuals: a cross-sectional study

**DOI:** 10.1186/s12995-015-0081-6

**Published:** 2015-10-26

**Authors:** Tiina Föhr, Asko Tolvanen, Tero Myllymäki, Elina Järvelä-Reijonen, Sanni Rantala, Riitta Korpela, Katri Peuhkuri, Marjukka Kolehmainen, Sampsa Puttonen, Raimo Lappalainen, Heikki Rusko, Urho M. Kujala

**Affiliations:** Department of Health Sciences, University of Jyväskylä, P.O. Box 35, FIN-40014 Jyväskylä, Finland; Methodology Centre for Human Sciences, Faculty of Social Sciences, University of Jyväskylä, P.O. Box 35 (Y 33), FIN-40014 Jyväskylä, Finland; Department of Psychology, University of Jyväskylä, P.O. Box 35, FIN-40014 Jyväskylä, Finland; Institute of Public Health and Clinical Nutrition, University of Eastern Finland, Kuopio Campus, P.O. Box 1627, FIN-70211 Kuopio, Finland; Medical Faculty, Pharmacology, Medical nutrition physiology, University of Helsinki, P.O. Box 63, FIN-00014 Helsinki, Finland; Finnish Institute of Occupational Health, Topeliuksenkatu 41 a A, FIN-00250 Helsinki, Finland; Institute of Behavioral Sciences, University of Helsinki, P.O. Box 9, FIN-00014 Helsinki, Finland; Department of Biology of Physical Activity, University of Jyväskylä, P.O. Box 35, FIN-40014 Jyväskylä, Finland

**Keywords:** Heart rate variability, Objective stress, Perceived stress scale, Physiological stress, Physical activity, Psychological stress, Recovery, Stress assessment, Subjective stress, Work-related stress

## Abstract

**Background:**

The present study aimed to investigate how subjective self-reported stress is associated with objective heart rate variability (HRV)**-**based stress and recovery on workdays. Another aim was to investigate how physical activity (PA), body composition, and age are associated with subjective stress, objective stress, and recovery.

**Methods:**

Working-age participants (*n* = 221; 185 women, 36 men) in this cross-sectional study were overweight (body mass index, 25.3–40.1 kg/m^2^) and psychologically distressed (≥3/12 points on the General Health Questionnaire). Objective stress and recovery were based on HRV recordings over 1–3 workdays. Subjective stress was assessed by the Perceived Stress Scale. PA level was determined by questionnaire, and body fat percentage was assessed by bioelectrical impedance analysis.

**Results:**

Subjective stress was directly associated with objective stress (*P* = 0.047) and inversely with objective recovery (*P* = 0.046). These associations persisted after adjustments for sex, age, PA, and body fat percentage. Higher PA was associated with lower subjective stress (*P* = 0.037). Older age was associated with higher objective stress (*P* < 0.001). After further adjustment for alcohol consumption and regular medication, older age was associated with lower subjective stress (*P* = 0.043).

**Conclusions:**

The present results suggest that subjective self-reported stress is associated with objective physiological stress, but they are also apparently affected by different factors. However, some of the found associations among these overweight and psychologically distressed participants with low inter-individual variation in PA are rather weak and the clinical value of the present findings should be studied further among participants with greater heterogeneity of stress, PA and body composition. However, these findings suggest that objective stress assessment provides an additional aspect to stress evaluation. Furthermore, the results provide valuable information for developing stress assessment methods.

## Background

Stress at work can be considered a major public health risk. In many theories and definitions, stress is determined as a psychological or physiological response of the organism to an external load [1]. Stress is associated with psychological disturbances, as well as physiological changes, such as lowered heart rate variability (HRV) [2]. It may even lead to cardiovascular diseases if prolonged [3, 4]. Recovery from stress is an important issue, as incomplete recovery is also suggested to be a risk factor for cardiovascular diseases [5]. Common to the definitions of recovery is that recovery occurs when the exposure to stress is over. Recovery repairs the negative effects of stress and it is a process during which individual recovers to the specific baseline level and is recovered from the possible previous loads [1]. Furthermore, stress has detrimental economic consequences due to decreased job satisfaction and increased sickness-related absences from work [6].

Most stress assessment methods primarily focus on an individual’s subjective perception of stress. However, HRV has been proposed to be a good indicator for investigating the physiological effects of stress and recovery [2, 7]. HRV is the beat-to-beat (R–R interval) variation in times between the consecutive heartbeats expressed in normal sinus rhythm on an electrocardiogram (ECG) recording [8, 9]. HRV is very individual and it is modulated by both parasympathetic and sympathetic activity of the autonomic nervous system (ANS). Stress is associated with increased activation level of the body when sympathetic activity dominates the ANS whereas recovery is associated with reduced activation level of the body when parasympathetic activation dominates the ANS over sympathetic activity [10–12]. Studies investigating the association between self-reported, psychological stress and HRV in real-life settings have found controversial results (e.g. [7, 13–16]). Most of these studies have used traditional time- or frequency-domain measures of HRV. However, it is also possible to describe the state of stress and recovery using HRV-derived variables that include information that is difficult to obtain from traditional measures of HRV [7]. These novel variables take into account factors such as HRV-derived respiratory variables and individual resting heart rate (HR) and HRV values. Due to very high inter- and intra-individuality of HRV, new approaches which take into account individuality in HRV could provide additional insight into quantification of stress and recovery.

Physical activity (PA) can help the individual to build resources to buffer the negative effects of stress and promote his/her recovery from stress [17]. On the other hand, stress is suggested to be associated with physical inactivity (e.g. [7, 18, 19]) and being overweight [7, 20]. High stress levels could attenuate an individual’s willingness or ability to engage in regular exercise and to be physically active [21], resulting in failure to achieve the beneficial effects of PA against stress.

The present study aimed to examine the associations between subjective self-reported psychological stress and objective physiological HRV-based measures of stress and recovery on workdays. The second aim was to investigate how PA, body composition, and age are associated with subjective stress, objective stress, and recovery.

## Methods

### Participants

The present cross-sectional study included individuals of different occupations with symptoms of metabolic syndrome and psychological distress, who met the inclusion criteria in an initial screening and underwent baseline measurements for a controlled and randomized trial called the Elixir study [22]. The initial purpose of the Elixir study was to investigate the effect of different psychological interventions on psychological and metabolic health. Data collection was carried out in three Finnish study centers at the universities of Helsinki, Jyväskylä, and Kuopio. The inclusion criteria were self-reported body mass index (BMI) of between 27–34.9 kg/m^2^, and perceived psychological stress indicated by at least 3/12 points on the General Health Questionnaire [23]. The participants did not have any severe chronic illnesses, and any regularly taken medications were reported. Details of the inclusion and exclusion criteria have been reported previously [22].

Further inclusion criteria specifically for the present secondary cross-sectional baseline analyses were available data regarding objective stress and recovery (HRV recording) and subjective stress (Perceived Stress Scale; PSS). Individuals who used α- or β-adrenergic blocking agents affecting the heart were excluded from the present analysis. However, other regular medication was allowed. The final study group of the present analyses comprised of 221 individuals who also met the additional inclusion and exclusion criteria. Table 1 presents the characteristics of the participants. All participants were informed about the initial study, and they signed written informed consent prior to any measurements. This study was conducted according to the Declaration of Helsinki, and the study protocol was approved by the ethics committee of the Central Finland Health Care District.

**Table 1:** Characteristics of the participants

	All (*N* = 221)		Female (*N* = 185)		Male (*N* = 36)	
	Mean	Range	Mean	Range	Mean	Range
Age (yrs)	48	26–60	48	26–60	45	31–60
Weight (kg)	87.7	64.0–120.1	85.4	64.0–113.9	99.3	83.8–120.1
Height (cm)	167.8	149.0–195.6	165.4	149.0–184.8	179.7	167.5–195.6
BMI (kg/[m]^2^)	31.1	25.3–40.1	31.2	25.3–40.1	30.8	26.3–37.0
Body fat%	38.6	12.8–50.8	40.7	28.4–50.8	28.0	12.8–35.1
Physical activity (MET index^a^)	3.2	0.0–18.0	3.0	0.0–15.3	4.3	0.1–18.0
Subjective stress (PSS^b^)	26.5	7.0–52.0	26.5	7.0–52.0	26.2	17.0–38.0
Objective stress (stress index)	163.0	88.5–455.8	163.2	88.5–455.8	162.2	89.1–308.3
Objective recovery (stress balance)	0.35	−1.00–1.00	0.34	−1.00–1.00	0.40	−0.98–1.00

^a^MET-h/day, based on retrospective physical activity questionnaire

^b^Perceived Stress Scale

### Measurements

Objective stress and recovery were determined from recordings of the beat-to-beat R–R interval in real-life settings over 1–3 workdays. R–R interval data were collected using a Firstbeat Bodyguard measurement device (Firstbeat Technologies Ltd, Jyväskylä, Finland). The data were then analyzed using the Firstbeat Analysis Server software (version 5.3.0.4), which included a powerful artifact correction feature for irregular ectopic beats, and signal noise. The original R-R interval series were resampled at the rate of 5 Hz by using linear interpolation to obtain equidistantly sampled time series, and second-by-second HRV indices were calculated with the short-time Fourier transform method by using constant duration Hanning window [24, 25]. Thereafter, the software categorizes the data into different physiological states such as stress, recovery, and physical activity of different intensities by taking into account individual characteristics (e.g. the individual levels and scales of HR and HRV, and the individual relationships between HRV and autonomic control e.g. [24]). Stress means sympathetic dominance of the ANS without metabolic requirements caused by physical activity whereas recovery means parasympathetic dominance of the ANS. In this categorization, second-by-second HRV indices, HRV-derived respiration rate, oxygen consumption calculated by HR, HRV-derived respiration rate, and on-off kinetics, and parameter describing excess-post exercise oxygen consumption are used with neural network data modeling [25–27] (for more details, see the white papers by Firstbeat Technologies Ltd [28]). The intensity of stress reaction is calculated from the HR, high frequency (0.15–0.4 Hz), and low frequency (0.04–0.15 Hz) components of HRV and respiratory variables. The intensity of stress is high when HR is elevated, HRV is reduced, and the frequency distribution of HRV is inconsistent because of changes in respiratory period. The intensity of recovery is calculated from the HR and high frequency component of HRV, and it is high when HR is low and high frequency component of HRV is high and regular. From the Firstbeat Analysis Server, the stress index was used as an indicator of objective stress and the stress balance value as an indicator of objective recovery. The stress index characterizes the magnitude of stress processes during the whole day. This index describes the mean intensity of the recognized stress reactions (theoretically ranging from 0 to ∞, ranging in our material 88.5 − 455.8). The stress balance value (ranging from −1 to 1) indicates the proportion of time of stress and recovery reactions during self-reported sleep periods during the measurement period. Values from 0.5 to 1 indicate good recovery, values from 0 to 0.5 indicate moderate recovery, and values from 0 to −1 indicate weak recovery [7].

The HRV data consisted of successfully recorded workdays, with an allowed maximum of 15 % regarding the grade of detected and corrected artifacts in R–R intervals. The values of the HRV-based variables of stress and recovery were the mean values of the workdays. Data from two workdays were included for 191 participants, from one workday for 20 participants, and from three workdays for 10 participants. For each monitored day, the participants reported their working hours, sleeping hours, and alcohol consumption in measurement diaries. Alcohol consumption was reported in standard units of approximately 12 g of ethanol (one unit: 33 centiliter [cl] beer, 12 cl red or white wine, 8 cl fortified wine, or 4 cl liquor).

Subjective stress during the preceding month was assessed using the 14-item PSS, which measures the degree to which situations in one’s life are stressful on a 5-point scale ranging from 0 (never) to 4 (very often) [29]. PSS total scores are generally calculated by reversing the scores of the seven positive items and then summarizing the scores of all 14 items. Internal reliability (Cronbach’s α) for the PSS was 0.86. To reduce the measurement error of the PSS sum score, we used a factor that included all 14 questions in the present analysis instead of the sum score.

PA was assessed using a questionnaire with items regarding present activity and changes within the last two months. The questionnaire included structured questions covering leisure-time PA and commuting activity [30, 31]. A multiple of the resting metabolic rate (MET) was assigned for each activity to describe the intensity of the form of PA. The MET indices for each form of PA were calculated by multiplying the intensity (MET), duration (h), and frequency of the activity, and the MET index was expressed as the sum score of different activities (MET-h/d).

Body composition was evaluated using bioelectrical impedance analysis (InBody720; Jyväskylä, Kuopio/Tanita BC-418 MA; Helsinki) in the morning after 10–12 h of fasting. This device provides information about the whole body fat percentage. Body weight and height were also measured during the same laboratory visit.

### Statistical analysis

Statistical analyses were performed using Mplus version 5.21 [32]. Within this program, we used the MLR estimator, which comprises maximum likelihood with robust standard errors and with scale-corrected chi-square test values correcting for the effect of non-normality. To analyze the measurement structure of the PSS score, we used confirmatory factor analysis (CFA). Based on the results of the CFA the latent factor model was used in the estimation of the correlations and in structural equation modeling analysis to investigate the associations of objective stress and recovery with subjective stress, as well as to investigate the associations of PA, body composition, and age with objective and subjective stress. The significance level of the study was set at 0.05. The model fit was evaluated using the *χ*^2^ test, comparative fit index (CFI), Tucker Lewis index (TLI), root mean square error of approximation (RMSEA), and standardized root mean square residual (SRMR). For a good-fitting model, the *χ*^2^ test is non-significant, CFI and TLI are at least 0.95, RMSEA is no more than 0.06, and SRMR no more than 0.08 [33].

## Results

**Fig. 1:**
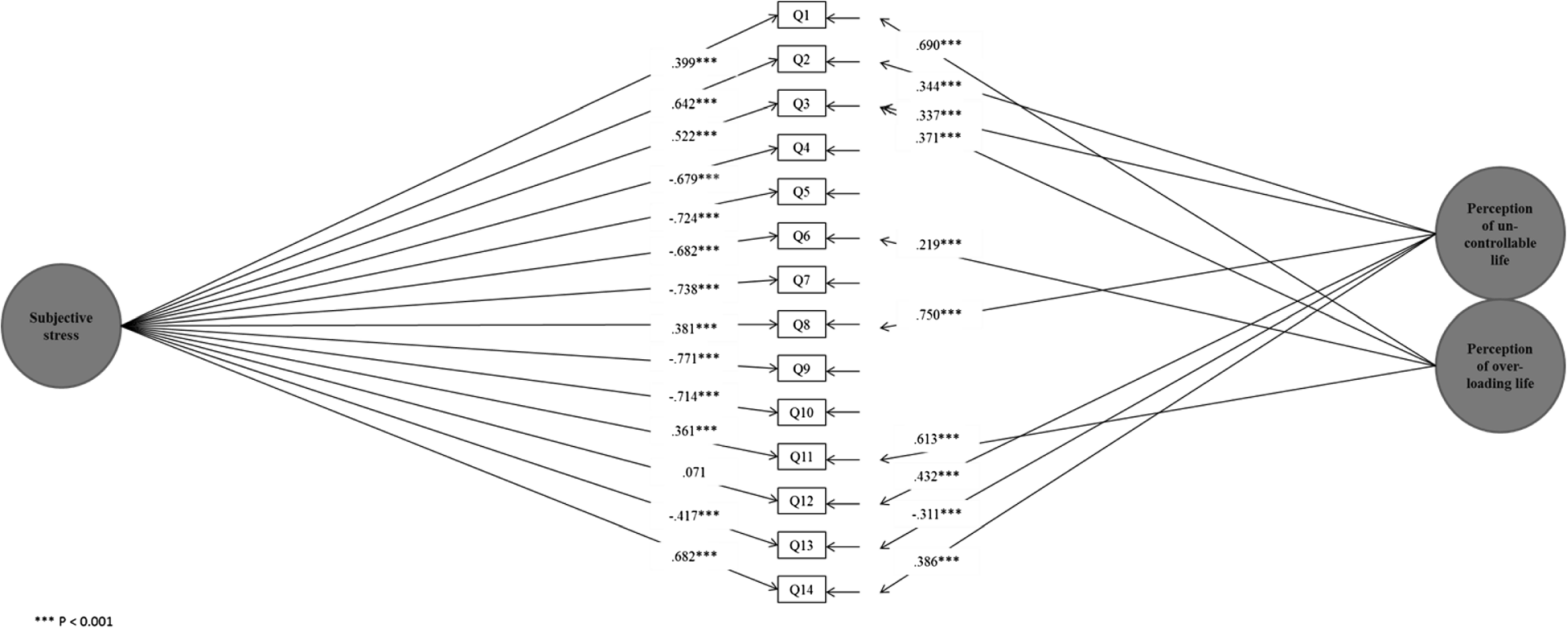
The structure of the factor constructed of the questions of the Perceived Stress Scale (PSS). Factor loadings (standardized estimates) for the 14 questions, and loadings of the two specific factors for residual variances

The one-factor solution for the PSS did not sufficiently fit the data (*χ*^2^ (77) = 345.12, *P* < 0.001; CFI = 0.786; TLI = 0.747; RMSEA = 0.126; SRMR = 0.084); therefore, the factor structure was altered based on modification indices. The factor subjective stress was merged to contain two specific factors, as covariance existed between the residuals of questions 2, 3, 8, 12, 13, and 14 (related to perception of uncontrollable life) and the questions 1, 3, 6, and 11 (related to perception of overload) (Fig. 1). This estimated model fit the data well (*χ*^2^ (66) = 109.25, *P* < 0.001; CFI = 0.966; TLI = 0.952; RMSEA = 0.054; SRMR = 0.044). All of the modification indices were lower than 4, indicating that there were no additional parameters that would have increased the fit of the model for the analysis.

Table 2 presents the results of the associations of subjective stress with objective stress and recovery. Subjective stress correlated with objective stress (*r* = 0.139, 95 % CI 0.002 to 0.276, *P* = 0.047) and with objective recovery (*r* = −0.140, 95 % CI −0.277 to −0.003, *P* = 0.046) without any adjustments (Model 1). In Model 2, the associations of subjective stress with objective stress and recovery were adjusted for sex and age. Subjective stress was associated with objective stress (residual *r* = 0.209, 95 % CI 0.075 to 0.343, *P* = 0.002) and with objective recovery (residual *r* = −0.137, 95 % CI −0.275 to 0.000, *P* = 0.050). Age was positively associated with objective stress (β = 0.435, 95 % CI 0.327 to 0.543, *P <* 0.001). Sex was not associated with subjective stress, objective stress, or recovery. The associations of subjective stress with objective stress and recovery were not influenced by further adjustment for alcohol consumption or for regular medication.

**Table 2:** Correlations of subjective stress with objective stress and recovery

	Subjective stress							
	Model 1				Model 2			
	r	S.E.	95 % CI	*P*	r_e_	S.E.	95 % CI	*P*
Objective stress (Stress index)	0.139	0.070	0.002 to 0.276	0.047	0.209	0.068	0.075 to 0.343	0.002
Objective recovery (Stress balance)	−0.140	0.070	−0.277 to −0.003	0.046	−0.137	0.070	−0.275 to 0.000	0.050

Standardized model results: correlation (r), residual correlation (r_e_), standard error (S.E.), 95 % confidence interval (95 % CI), and *P* value

Model 1: no adjustments Model 2: age- and sex adjusted

**Fig. 2:**
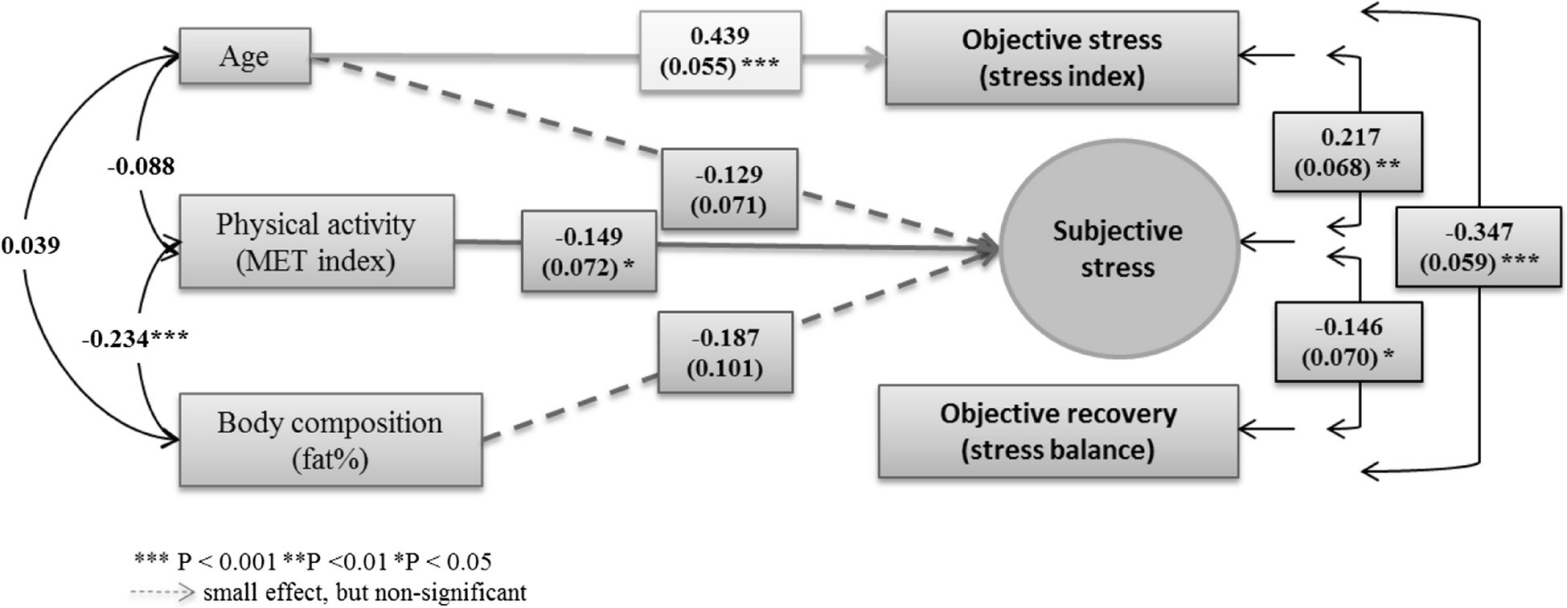
Sex-adjusted associations of age, physical activity, and body composition with subjective stress, with objective stress, and with recovery. The presented values are standardized model results (estimate and standard error). Residual correlation and standard error between subjective stress and objective stress and recovery, and correlations between age, physical activity, and body composition are also presented

Figure 2 presents the association of subjective stress with objective stress and recovery after adjustment for sex, age, PA, and body fat percentage. Subjective stress was associated with objective stress (residual *r* = 0.217, 95 % CI 0.084 to 0.351, *P* = 0.001) and recovery (residual *r* = −0.146, 95 % CI −0.283 to −0.009, *P* = 0.037). PA was negatively associated with subjective stress (β = −0.149, 95 % CI −0.289 to −0.009, *P* = 0.037). The negative association of subjective stress with body fat percentage was non-significant (β = −0.187*,* 95 % CI −0.384 to 0.011, *P* = 0.064). Objective stress and recovery were not associated with PA or body fat percentage. Sex was not associated with subjective stress, objective stress, or recovery. Age was positively associated with objective stress (β = 0.439, 95 % CI 0.330 to 0.547, *P* < 0.001). The results were not influenced by further adjustment for alcohol consumption or regular medication, except that age was negatively associated with subjective stress (β = −0.143, 95 % CI −0.282 to −0.004, *P* = 0.043).

## Discussion

The present findings suggest that the higher the subjective self-reported stress the higher the objective HRV-based stress and the lower the objective HRV-based recovery on workdays. We also found that within our psychologically distressed and overweight study group, higher level of PA was associated with lower subjective stress.

Our results showed that higher subjective stress was associated with higher objective stress when adjusted for the effects of age and sex. This rather weak but statistically significant association strengthened after accounting for PA and body fat percentage. This finding is in line with those of previous studies. Uusitalo et al. [34] found that daily emotions at work and chronic work-related stress were associated with cardiac autonomic function. They used the same HRV-based measurement method as we did, and reported that the associations of HRV-based measures and the traditional HRV measures with subjective stress were alike. Other previous studies have reported similar findings using traditional time or frequency domain measures of HRV (e.g. [14, 16]). Collins et al. [14] observed that job strain and low decision latitude were associated with reduced cardiac vagal control, and that job strain was associated with elevated sympathetic control during working hours. These results of Collins et al. [14] were obtained from 24-h ECG recordings. Additionally, other study results obtained from both short-time [35, 36] and 24-h ECG recordings (e.g. [37]) have observed the association between subjective stress and HRV with larger populations.

We also found that higher subjective stress was associated with lower objective recovery during sleep. This rather weak but statistically significant association strengthened after taken into account sex, age, PA and body fat percentage. This finding supports the previous study, which found that daily emotions at work were associated with night time traditional HRV measures [34]. In contrast, Hynynen et al. [13] found that low- and high-stress groups exhibited no differences in nocturnal traditional measures of HRV. Further studies are needed to investigate the association between subjective stress and HRV-based recovery during sleep. Recovery is an important factor in preventing the detrimental effects of stress, as good recovery during sleep has been suggested to confer protection from cardiovascular disease risk factors [2, 38]. An effort-recovery model shows that in cases of incomplete recovery, an employee must expend extra effort to perform properly at work [1].

The present results showed that higher level of PA was weakly but statistically significantly associated with lower levels of subjective stress among our overweight participants with low inter-individual variability in PA. Within our study group, PA was not associated with objective stress or objective recovery. The present study design does not enable us to draw any broader conclusions about the association between PA and subjective stress, and it remains unclear whether stress or inactivity is a cause or a consequence in this relationship. However, our finding of a negative association between PA and subjective stress supports the conclusions of previous studies. Hamer [39] suggests that regular exercise may have stress-buffering benefits largely because exercisers are more often in the post-exercise window when they encounter daily stressors. In line with our present cross-sectional finding, Kouvonen et al. [18] found that increased work-related stress was weakly associated with decreased PA during 6–8 year follow-up. In contrast to our present results, we found in our previous study [7] that a higher level of PA was associated with lower objective stress and higher objective recovery among participants with great inter-individual variability in their PA levels.

Within our study group, body fat percentage was not associated with objective stress or recovery. According to magnitude-based inference with confidence intervals [40, 41] our results showed a small negative effect of body fat percentage on subjective stress. In line with this, previous findings in large study groups suggest that a higher obesity level may confer protection from burnout (e.g. [42]). Whereas, our previous study found that a more favorable body composition was associated with lower objective stress and higher objective recovery [7] and obesity is known to cause stress to the body, as visceral obesity is a key factor in metabolic syndrome [43].

Here we also found that older age was associated with higher objective stress, but not with objective recovery. Additionally, the results showed an unclear negative non-significant association between age and subjective stress. Our inference is that any effect of age on subjective stress is at most small. HRV is individual, and is known to decrease with age [11]. The objective method presently used recognizes stress and recovery reactions and determines different physiological states of the body based on individual scaling of HR and HRV data. The presently used stress index as an indicator of magnitude of the objective stress reactions is, however, calculated from HR, HRV and respiratory variables during the recognized stress states on the whole day. Therefore, the aging-related decrease in HRV likely explains this finding to some extent and inhibits us to conclude that older employees were more sensitive to physiological stress. Whereas, the presently used stress balance value as an indicator of objective recovery was calculated from the recognized stress and recovery states during sleep, and this value takes into account all individuality in HRV. In the present study, age did not affect objective recovery during sleep. However, older persons seemed to perceive less stress than their younger counterparts. Further research on the association between age and objective stress, including the effect of PA, would be interesting and warranted.

In the present study, subjective stress was assessed by PSS. This scale has been found to be a reliable and valid measure of perceived stress. To analyze the measurement structure of the PSS score, we further used CFA to test whether the 14 questions of the PSS were consistent with the factor consisting of these 14 questions. We found that the fit factor solution included two specific factors. Based on the results of the CFA and to get more reliable results we used this factor solution as a variable of subjective stress in the present analysis. PSS assesses subjective stress during the preceding month. So, it is important to take into account that the present results provide information about the association of perceived stress over one month with the objective stress and recovery at the end of this month.

Subjective methods were used to assess both the level of stress and the level of PA. Even though, it is known that subjective assessment may include some reporting bias, it is a commonly used method for measuring stress. The most frequently utilized instrument is a questionnaire that has been widely criticized because it relies on individual self-reporting. In contrast, physiological measures represent objective data, which are considered to be more reliable as they are not affected by an individual’s cognition, social context, or emotions [44]. Therefore, it is reasonable and important to develop objective measures of stress that acknowledge the physiological burden of stress. The assessment of PA was based on a questionnaire. However, this questionnaire took into account a long-term level of PA and may give more trustful information about individual’s PA than an objective measurement from a short period.

HRV assessment is a promising clinical tool for assessing health and identifying health impairments [10, 24, 45]. However, due to a limited applicability of the traditional measures of HRV into changing and uncontrollable real-life setting, it is important to develop methods that apply HRV data and are able to provide valuable information on stress and recovery in individuals’ everyday life. By utilizing both HRV and additional HRV-derived information it is possible to produce knowledge of physiological states, such as stress and recovery that is not available from the basic HRV measures which represent the average level of the autonomic activity over a period of the time. This kind of information can also be utilized in supporting lifestyle change. Previous findings also support the validity and reliability of the HRV-based method used in the present study [7, 34]. In addition to subjective assessment of stress, the method has been validated against neuroendocrine responses to stress. The indicators of stress and recovery during sleep have been found to be associated with free salivary cortisol after awakening [46]. As HRV measurement is dependent on the duration of the R − R interval recording [47], it is a strength of the present study that HR and HRV data were recorded in real-life settings over a rather long time period, usually two workdays. Secondly, the method produced stress- and recovery-related variables based on the HR, HRV, and respiratory variables. These variables are more informative for both health-care professionals and patients/clients than traditional measures of HRV and they are suitable for application in general health care.

Although, HRV assessment is a promising clinical tool for assessing health and identifying health impairments, several points must be considered regarding the HR- and HRV-based measurement of stress. HR and HRV are very individual, and are dependent on age and sex [10, 11, 24]. HRV decreases with age, and the variation is greater among female population. Therefore, it is important to account for age and gender, as well as PA, when investigating the associations of objective stress and recovery with subjective stress. The present study excluded subjects who used α- or β-blockers, as these drugs have substantial effects on HR and HRV. However, our participants included individuals using regular medication such as other cardiac medications or psychopharmacological, metabolic or analgesic medication. This could also potentially affect HR and HRV since many medications directly or indirectly act on the ANS [11]. Therefore, we also accounted for regular medication and alcohol consumption in our present analysis. These adjustments did not affect the associations of objective stress and recovery with subjective stress. However, it is important to acknowledge that the measurement was accomplished in real-life settings and it takes participant’s word for confounding factors, such as medication and alcohol consumption.

The present study participants were psychologically distressed and overweight composing an ideal study group for investigating the association of subjective stress with objective stress and recovery. Furthermore, they represent a group of Finnish workers of different occupations, mainly trade and office workers. However, there were limited variations in the levels of psychological stress, fat percentage and PA within our participants. Therefore, these presently found associations should be studied further among more heterogeneous study group and the present results can thus be generalized to overweight individuals with psychological distress. It would have been interesting to further study the possible gender differences; however, such analyses were not reasonable within the present group due to the low number of men. Data collection was carried out in three study centers. However, adjustment for study center did not affect the results.

There is a recognized need for further research about the reliability of HRV measurement. The results of Cipryan & Litschmannova [48] suggest that researchers should be very cautious when drawing conclusions based only on short-term HRV analysis. Such methods as presently described and evaluated HRV-based method for assessing objective stress and recovery in real-life settings mainly over two workdays, are necessary to improve the reliability of HRV analysis and to provide informative and suitable variables for application in general health care and lifestyle counselling. This kind of individual counselling would be the optimal way to utilize HRV-based method in stress assessment. Real-life setting includes several uncontrollable confounding factors complicating the group-level analyzing of the data. This fact together with the participants’ low inter-individual variation in the main variables of the present study result to the fact that the found associations are rather weak. From the perspective of magnitude-based inference with confidence intervals [40] there is a possibility that some of our findings are somewhat trivial. On the whole, there is a limited number of studies that have utilized long-term HRV monitoring and it is difficult to compare the present findings with the previous findings obtained from short-term ECG recordings and with studies that have used different measures than we did. Nevertheless, based on previous evidence, we assume that similar findings to our present findings and overall conclusion would have been reached by using traditional measures of HRV. More research with larger study populations and further follow-up studies are needed.

## Conclusions

The present results suggest that subjective self-reported stress is associated with objective HRV-based stress and recovery. Subjective psychological and objective physiological stress are apparently affected by different factors, such as PA. However, some of the found associations among the overweight and psychologically distressed participants with low inter-individual variation in PA are rather weak and the clinical value of the present findings should be studied further among participants with greater heterogeneity of stress, PA and body composition. However, these findings suggest that the presently described method for objective stress assessment provides an additional and important aspect to stress assessment. Furthermore, the results provide valuable information for the development of stress assessment methods.

## Abbreviations

ANS: Autonomic nervous system
BMI: Body mass index
CFA: Confirmatory factor analyses
CFI: Comparative fit index
ECG: Electrocardiogram
HR: Heart rate
HRV: Heart rate variability
MET: Multiple of the resting metabolic rate
PA: Physical activity
PSS: Perceived stress scale
RMSEA: Root mean square error of approximation
SRMR: Standardized root mean square residual
TLI: Tucker Lewis index
